# Supplementary material for: A novel customer churn prediction model for the telecommunication industry using data transformation methods and feature selection
Source: PLoS One. 2022 Dec 1;17(12):e0278095. doi: 10.1371/journal.pone.0278095 (PMC9714823; doi:10.1371/journal.pone.0278095)
Supplement: Attachment — Submitted filename: ReviewResponse.pdf [file pone.0278095.s003.pdf]

Response to Reviewer Comments

Reviewer #1:

The paper proposed a prediction model for the telecommunication industry using data transformation methods and feature selection; however, the article should be revised as follows:

1.English writing is good but can be improved by a native.

Author response: We thank you for this valuable feedback. In this updated draft, we believe the write up has been improved significantly. In addition to thorough review by the first author, a software has been used to check for grammar errors, literary styles etc. Last but not least two of the co-authors, including the corresponding author, are fluent English speakers. The corresponding author, while not a native of any English speaking country, has lived in the United States for over 7 years and thus gained the ability to fluently and eloquently communicate in English, like a native speaker. He has thoroughly reviewed the manuscript, made necessary editorials to improve the eloquence of the manuscript. We sincerely hope that the write up has a smooth flow now.

2.The abstract should be re-written, and principal research gaps and contributions are unclear.

Author response: We thank the reviewer for this feedback. We have updated our abstract to mention that our literature review has clearly exposed room for improvement in the field of customer churn prediction in the telecommunication industry. In Section 1.1 under the Introduction section, we have specifically called out these research gaps. A table (Table 1) has also been added to further specify the limitations of the previous studies. In Section 1.2, we have articulated our contributions towards closing these gaps. We believe this updated manuscript addresses the reviewer's concern adequately.

3. Although the introduction is well-organized, the existing research gaps were not properly discussed and listed in the introduction section.

Author response: Thank you for the feedback. Please see our response to the previous feedback, which should address this concern as well.

4. The work's main contributions and novelty can be re-written and focus mostly on the novelties.

Author response: We thank the reviewer for this feedback. We acknowledge that in the previous version of the manuscript we were not crisp enough in calling out the main contributions and novelties of our work. Based on your feedback, in this updated manuscript, we have specifically called out the key contributions in this work in *Section 1.2 Out Contributions.* Besides, in Table

1, we have listed the data transformation (DT) methods that were or were not used in previous studies. We have explored several DT methods which have never been used in the context of customer churn prediction (CCP) problems in the telecommunication industry (TCI). This adds to the novelty of our work. Similarly, the notion of hyperparameter optimization and feature selection has mostly been ignored in prior literature of CCP in TCI problem. In this study, we have introduced hyperparameter tuning as well as feature selection in this context.

5. In the Tables, the best-found results can be bold.

Author response: We thank the reviewer for this very valuable feedback. The best results are now shown in bold-face in Tables 10, 11, 12, 13.

6. Please develop the section of the related works separately, and develop the current literature review using some references about the hyper-parameters tuning of deep learning models such as: a) A deep learning-based evolutionary model for short-term wind speed forecasting: A case study of the Lillgrund offshore wind farm. Energy Conversion and Management, 236, 114002. b) Short-term wind speed forecasting using recurrent neural networks with error correction, Energy, Volume 217, 2021, 119397. c) LSTM based long-term energy consumption prediction with periodicity, Energy, Volume 197, 2020. d) Prediction of electricity generation from a combined cycle power plant based on a stacking ensemble and its hyperparameter optimization with a grid-search method, Energy, Volume 227, 2021.

Author response: We thank the reviewer for this feedback. In Section 2.6 of the revised manuscript, we have discussed the related work of hyperparameter optimization. We thank the reviewer for suggesting some papers. We have reviewed the papers and referred the ones that are closely related to our study.

7. What are the benefits and drawbacks of grid search method? please add them.

Author response: Thank you for this feedback. We have discussed the benefits and drawbacks of the grid search method in Section 2.6 of the revised manuscript.

8. The applied and optimised hyper-parameters should be listed in a table.

Author response: Thank you for this suggestion. Accordingly, we have listed the applied and optimized hyperparameters in Table 5 of the revised manuscript.

9. If it is possible provide a 3-D plot of the grid search performance for hyper-parameters tuning

Author response: In Figure 2 of the revised manuscript, we have depicted how the performance of the predictors varied with the search for the optimal hyperparameters for logistic regression

(LR) and feed-forward neural network (FNN). For brevity, we did not produce curves for all classifiers. For logistic regression, the parameter C has been tuned. The best performance was attained when C = 5. In the case of FNN, the hyperparameters epoch and batch_size were optimized. The best performance is attained when epoch = 10, batch size = 10.

Reviewer #2:

1) Customer churn prediction model for telecom using machine learning technique is not a new concept. Hence, it is not convinced that the model is novel and novelty of the model needs to be well demonstrated.

Author response: We thank the reviewer for this feedback. We acknowledge that in the previous version of the manuscript we were not crisp enough in calling out the main contributions and novelties of our work. Based on your feedback, in this updated manuscript, we have specifically called out the key contributions in this work in *Section 1.2 Out Contributions.* Besides, in Table 1, we have listed the data transformation (DT) methods that were or were not used in previous studies. We have explored several DT methods which have never been used in the context of customer churn prediction (CCP) problems in the telecommunication industry (TCI). This adds to the novelty of our work. Similarly, the notion of hyperparameter optimization and feature selection has mostly been ignored in prior literature of CCP in TCI problem. In this study, we have introduced hyperparameter tuning as well as feature selection in this context.

2) The organization of the manuscript should be mentioned in Introduction.

Author response: We thank the reviewer for this feedback. We have mentioned the organization in the Section 1.3 in the revised manuscript.

3) The literature review should emphasize both the findings and limitations. It is better to produce a comparative table.

Author response: We thank the reviewer for this wonderful suggestion. In the revised manuscript, we have discussed the findings and limitations of prior works in Section 1.1. We have also added a table (Table 1) that compares various previous studies in terms of use of DT methods, hyperparameter tuning and feature selection.

4) The optimization of the machine learning classifiers is not well demonstrated.

Author response: We have mentioned the optimization issues in Section 2.6 in the revised manuscript. We have articulated the hyperparameter values used in the various classifiers in Table 5.

5) Authors should provide more precise and critical comparison on existing related works. Need to provide more details on what is the research gap in the existing machine learning model and what are the possible ways to improve those.

Author response: Thank you for this valuable feedback. In the revised manuscript, we have provided the comparison of existing related works in Section 1.1 and Table 1. In Section 1.1 we have also discussed the existing research gaps and ways to tackle those gaps. We have also compared our models with CCP models from prior works in Table 9 of this revised manuscript..

6) Please revise all of the English. It is very important that the manuscript is finally revised by a native speaker.

Author response: We thank the reviewer for this valuable feedback. In this updated draft, we believe the write up has been improved significantly. In addition to thorough review by the first author, a software has been used to check for grammar errors, literary styles etc. Last but not least two of the co-authors, including the corresponding author, are fluent English speakers. The corresponding author, while not a native of any English speaking country, has lived in the United States for over 7 years and thus gained the ability to fluently and eloquently communicate in English, like a native speaker. He has thoroughly reviewed the manuscript, made necessary editorials to improve the eloquence of the manuscript. We sincerely hope that the write up has a smooth flow now.

Reviewer #3:

• The abstract section is very generalized and cannot reveal the clear outcomes of the proposed study.

Author response: We thank the reviewer for this comment. We have updated the abstract section in our revised manuscript to articulate the outcomes of this study.

• The literature is outdated because there is a need to cite articles from 2019 and onward.

Author response: Thank you for this valuable feedback. Accordingly we have updated our literature review by citing a few recent references (ref 1, 5, 7, 31, 33, 35, 39, 42).

• The proposed study may also clearly distinguish the work presented in this article from existing work https://www.sciencedirect.com/science/article/abs/pii/S0268401218305930

Author response: We thank the reviewer for this very important feedback. Accordingly we have dedicated a separate paragraph in *Section 1.1. Brief Literature review* to make distinction between that study and ours. Amin et al. have experimented with only 1 dataset for model training and another dataset for independent testing. The training set is much smaller compared to the largest dataset (Dataset 1) that we have used in our study. Not surprisingly, the models

trained in this dataset perform significantly better compared to their models. Besides, their study did not consider the WOE DT method, which has been shown to be the best performing DT method in the majority of the cases in our experiments. We have also experimented with a more comprehensive list of baseline classifiers compared to their study.

• The authors have used three datasets and provided the following source links:

1. https://www.kaggle.com/abhinav89/telecom-customer/data (Last Access: September 29, 2019)

2. https://data.world/earino/churn (Last Access: February 10, 2020)

3. https://www.kaggle.com/becksddf/churn-in-telecoms-dataset/data (Last Access: February 10, 2020)

However, I have observed that URL-2 and URL-3 are the same datasets. The number of samples of both datasets is different. One dataset contains 3333 samples, and the second dataset has 5000 samples. I will recommend to considered different datasets.

Author response: We thank the reviewer for this feedback. We agree that both the datasets have the same set of features. However, as the sample size is different we have to treat them as separate datasets and accordingly report our results. However, based on your feedback, we have added yet another dataset (Dataset 4) in our experiments. The results in this dataset has been presented in Section 3.4 and Table 13.

• I have a few observations on figure-1, which is the proposed flowchart of the optimized CCP model:

1. What is done during the preprocessing step may also be illustrated in the preprocessing block?

Author response: Thank you for your feedback. We have modified Figure 1 in the revised manuscript. We have illustrated in detail in the data preprocessing block.

2. Why specifically used univariate feature selection?

Author response: We thank the reviewer for this question. We have discussed the benefit of the univariate feature selection method in Section 2.6 in our revised manuscript.

3. Why straightaway terminate the process after 10-fold validation? I think 10-fold validation will produce some results which may be calculated using evaluation measures and will be used for comparison of Machine learning methods.

Author response: Thank you very much for this observation. Based on this feedback, in our revised manuscript, we have added the evaluation measure step in Figure 1.

4. It would be more appropriate if you could add a statistical test or significance test, which is currently missing.

Author response: Thank you. We have already conducted a statistical test (Freedman test) which is presented in the Section 2.7 (Stability measurement tests).

Reviewer #4:

The contribution of this paper is good and I am happy to endorse its acceptance at some point. However, there are several major and minor comments to address. I have listed them as follows:

Please clearly state the gap targeted in this paper at the end of introduction and list down the hypotheses.

Author response: We thank you for your kind observation. In this updated manuscript, we have specifically called out the key contributions in this work in *Section 1.2 Out Contributions.* Besides, in Table 1, we have listed the data transformation (DT) methods that were or were not used in previous studies. The notion of hyperparameter optimization and feature selection has mostly been ignored in prior literature of CCP in TCI problem. In this study, we have introduced hyperparameter tuning as well as feature selection in this context.

In terms of research method and design, there is not much in the paper.

The comparative algorithms in the experiments are not properly acknowledged and cited.

Author response: Thank you for your feedback. In this updated manuscript, we have made several updates in the description in the *Materials and Methods* section. We have also added a few recent references (2019 - 2022). We have added a comparative study with prior works in Section 4 and Table 9, with appropriate citations. We believe that we have been able to incorporate your feedback in this revised manuscript.

I also suggest adding some figures to better articular the content as the paper looks very dry at the moment.

Author response: Thank you. We have added a new figure (Figure 2) in the context of hyperparameter optimization in the revised manuscript.

Analysis of the results is missing in the paper.

There is a big gap between the results and conclusion. There should be the result analysis between these two sections. After comparing the numerical methods, you have to be able to analyse the results and relate them to their structures. It would be interesting to have your thoughts on why the method works that way? Such analyses would be the core of your work

where you prove your understanding of the reason behind the results. You can also link the findings to the hypotheses of the paper. Long story short, this paper requires a very deep analysis from different perspectives.

Author response: We thank the reviewer for this feedback. We have made several updates throughout the manuscript to improve the presentation of our work. We have added analysis on a 4th dataset, the result of which has been added in Section 3.4. We also have a Discussion section (Section 6) in our manuscript where we discuss (among other points), we certain aspects of our results are different in the different datasets. Overall, we believe we have been able to provide a deep and thorough analysis of our experimental results.

There is no statistical test to judge about the significance of the numerical method's results. Without such a statistical test, the conclusion cannot be supported.

Author response: We thank the reviewer for this feedback. However, we had already conducted a statistical test (Freedman test) which is presented in the Section 2.7 (Stability measurement tests).

There is no discussion on the cost effectiveness of the proposed method. What is the computational complexity? What is the runtime? Please include such discussions. You can also use the big oh notation to show the computation complexity. Some mathematical notations and Lemma presentations are not rigorous enough to correctly understand the contents of the paper.

The authors are requested to recheck all the definition of variables and further clarify these equations.

Author response: We thank the reviewer for this feedback. We have discussed the running time of the grid search method for hyperparameter tuning in Section 2.6 and Table 6. The mathematical notations in Table 3 have been thoroughly reviewed and revised as needed.
