# Supplementary material for: A novel customer churn prediction model for the telecommunication industry using data transformation methods and feature selection
Source: PLoS One. 2022 Dec 1;17(12):e0278095. doi: 10.1371/journal.pone.0278095 (PMC9714823; doi:10.1371/journal.pone.0278095)
Supplement: Attachment — Submitted filename: ReviewResponse.pdf [file pone.0278095.s004.pdf]

Response to Reviewer Comments

Reviewer #4: Some final cosmetic comments::

The paper proposed a prediction model for the telecommunication industry using data transformation methods and feature selection; however, the article should be revised as follows:

1.The results of your comparative study should be discussed in-depth and with more insightful comments on the behaviour of your algorithm on various case studies. Discussing results should not mean reading out the tables and figures once again..

Author response: We thank you for this valuable feedback. In Sections 4 & 6 of the revised manuscript, we have discussed the comparative study in depth and more insightful comments have been included.

2.Avoid lumping references as in [x, y] and all other. Instead summarize the main contribution of each referenced paper in a separate sentence. For scientific and research papers, it is not necessary to give several references that say exactly the same. Anyway, that would be strange, since then what is innovative scientific contribution of referenced papers? For each thesis state only one reference.

Author response: We thank the reviewer for this feedback. In the revised manuscript, we have avoided lumping references as much as possible. In some cases, however, we felt that it was necessary to provide multiple references from literature to strengthen a claim. In such cases, we decided to stick with multiple references.

3. Avoid using first person..

Author response: Thank you for this suggestion. In this updated manuscript, we have avoided using first person whenever possible. However, on several occasions, we found that to maintain the flow of the narrative in an eloquent manner, the use of first person was necessary. In such cases we have kept the first person narrative..

4.Avoid using abbreviations and acronyms in title, abstract, headings and highlights.

Author response: We thank you for your kind observation. Accordingly, we have updated the manuscript.

5. Please avoid having heading after heading with nothing in between, either merge your headings or provide a small paragraph in between...

Author response: Thank you very much for this feedback. In this revised manuscript, we have added a few texts after the heading Section 2. We believe this was the only place where we had a heading after heading with nothing in between.

6.The first time you use an acronym in the text, please write the full name and the acronym in parenthesis. Do not use acronyms in the title, abstract, chapter headings and highlights.

Author response: We thank the reviewer for this comment. We have updated the manuscript Accordingly.

7. The results should be further elaborated to show how they could be used for the real applications.

Author response: Thank you for this suggestion. We have updated the Discussion section (Section 6) accordingly.
