# Supplementary material for: A novel customer churn prediction model for the telecommunication industry using data transformation methods and feature selection
Source: PLoS One. 2022 Dec 1;17(12):e0278095. doi: 10.1371/journal.pone.0278095 (PMC9714823; doi:10.1371/journal.pone.0278095)
Supplement: Attachment — Submitted filename: PLOS ONE data transformation paper.docx [file pone.0278095.s002.docx]

Article Title:

A novel customer churn prediction model for the telecommunication industry using data transformation methods and feature selection

Manuscript: PONE-D-22-11288

Reviewer's Comments:

In this article, the authors have conducted a comparative study on various data transformation methods (RAW, Log, Box-cox, Rank, Discretization, Z-score, WOE) followed by feature selection (univariate feature selection, etc.). Further, they have performed hyperparameter tuning for various machine learning methods (KNN, NB, LR, RF, DTree, GB, FNN, and RNN). However, the article's contents in its current state led to an emphasis on work presentation and some technical issues listed below.

- The abstract section is very generalized and cannot reveal the clear outcomes of the proposed study.
- The literature is outdated because there is a need to cite articles from 2019 and onward.
- The proposed study may also clearly distinguish the work presented in this article from existing work <https://www.sciencedirect.com/science/article/abs/pii/S0268401218305930>
- The authors have used three datasets and provided the following source links:
  1. https://www.kaggle.com/abhinav89/telecom-customer/data (Last Access: September 29, 2019)
  2. https://data.world/earino/churn (Last Access: February 10, 2020)
  3. https://www.kaggle.com/becksddf/churn-in-telecoms-dataset/data (Last Access: February 10, 2020)

However, I have observed that URL-2 and URL-3 are the same datasets. The number of samples of both datasets is different. One dataset contains 3333 samples, and the second dataset has 5000 samples. I will recommend to considered different datasets.

- I have a few observations on figure-1, which is the proposed flowchart of the optimized CCP model:
  1. What is done during the preprocessing step may also be illustrated in the preprocessing block?
  2. Why specifically used univariate feature selection?
  3. Why straightaway terminate the process after 10-fold validation? I think 10-fold validation will produce some results which may be calculated using evaluation measures and will be used for comparison of Machine learning methods.
  4. It would be more appropriate if you could add a statistical test or significance test, which is currently missing.
